# Psychiatric outpatient consultation for seniors. Perspectives of family physicians, consultants, and patients / family: A descriptive study

**DOI:** 10.1186/1471-2296-6-15

**Published:** 2005-04-19

**Authors:** Mark J Yaffe, Francois Primeau, Jane McCusker, Martin G Cole, Eric Belzile, Nandini Dendukuri, Michel Elie, Johanne Laplante

**Affiliations:** 1 Departments of Family Medicine, McGill University and St. Mary's Hospital Centre, 3830 Lacombe Avenue, Montreal, Qc, H3T 1M5, Canada; 2 Department of Psychiatry, Laval University, Quebec City, Qc, Canada; 3 Departments of Epidemiology and Biostatistics, McGill University and St. Mary's Hospital Centre, Montreal, Qc, Canada; 4 Department of Psychiatry, McGill University and St. Mary's Hospital Centre. Montreal, Qc, Canada; 5 Department of Clinical Epidemiology and Community Studies, St. Mary's Hospital Centre, Montreal, Qc, Canada; 6 Department of Epidemiology and Biostatistics, McGill University, Montreal, Quebec, Canada; 7 Department of Psychiatry, McGill University and St. Mary's Hospital Centre, Montreal, Qc, Canada; 8 Johanne Laplante, Departments of Nursing and Psychiatry, St. Mary's Hospital Centre, Montreal, Quebec, Canada

## Abstract

**Background:**

Family practitioners take care of large numbers of seniors with increasingly complex mental health problems. Varying levels of input may be necessary from psychiatric consultants. This study examines patients'/family, family practitioners', and psychiatrists' perceptions of the bi-directional pathway between such primary care doctors and consultants.

**Methods:**

An 18 month survey was conducted in an out-patient psychogeriatric clinic of a Montreal university-affiliated community hospital. Cognitively intact seniors referred by family practitioners for assessment completed a satisfaction and expectation survey following their visits with the psychiatric consultants. The latter completed a self-administered process of care questionnaire at the end of the visit, while family doctors responded to a similar survey by telephone after the consultants' reports had been received. Responses of the 3 groups were compared.

**Results:**

101 seniors, referred from 63 family practitioners, met the study entry criteria for assessment by 1 of 3 psychogeriatricians. Both psychiatrists and family doctors agreed that help with management was the most common reason for referral. Family physicians were accepting of care of elderly with mental health problems, but preferred that the psychiatrists assume the initial treatment; the consultants preferred direct return of the patient; and almost 1/2 of patients did not know what to expect from the consultation visit. The rates of discordance in expectations were high when each unique patient-family doctor-psychiatrist triad was examined.

**Conclusion:**

Gaps in expectations exist amongst family doctors, psychiatrists, and patients/family in the shared mental health care of seniors. Goals and anticipated outcomes of psychogeriatric consultation require better definition.

## Background

Concerns about both the nature and the adequacy of the provision of mental health services in primary care settings have prompted exploration of the respective roles of psychiatrists and family practitioners in such activities. The interface between physician groups has been examined from the perspective of family doctors gatekeeping access to specialized services in Canada [1], the United States [2], Britain [3], and Australia [4]. Attention has focussed on the specific purpose, role and outcome of the consultation process. While some motivation for these reviews was economic, the process and quality of communication between referring physician and consultant also began to be explored.[5] Goldberg and Huxley [6,7] have described potential obstacles along a pathway to mental health consultation, indicating the importance of integration of services from family doctor to specialty care. [8]

Within the domain of mental health services a shared care model between psychiatrist and family physician has been described [9,10] and advocated for the care of elderly.[11] For example, while the prevalence of major depression in an older primary care population is 6%, [12] less than 20% of these depressed elderly are diagnosed and adequately treated.[13] Interventions may be designed to improve the process of out-patient psychogeriatric care.[14] The PROSPECT [15,16] and IMPACT [17-20] studies have recently demonstrated, for example, the particular benefit of psychiatric care managers in managed health care settings.

Elderly with mental health problems usually present to primary care doctors [21] and the actual pathway to consultative specialized psychiatric services is complex.[13] Nutting et al distinguish between consultation, referral, and transfer of care.[22] The consultation involves one physician asking another to perform a specific diagnostic or therapeutic task, and to provide specific impressions and recommendations for care. In a referral the patient will see another physician for diagnosis, investigation and possible short term management of a specific problem, but continue to see the referring doctor for other issues. If a problem is complex there may be complete transfer for on-going care of the specific problem under review.

Problems in communication between family practitioners and psychiatrists may exist within the aforementioned options for care.[23] A referring note may not succinctly indicate why the input of a specialist is desired and what is specifically expected from the consultation. The consultation report may not respond with sufficient content to provide the family doctor with the necessary information to permit comfort to assume the care of the patient. In this regard Craven and Bland's overview of shared mental health services suggests that more information is required about why family doctors refer to or consult psychiatrists and whether the process is effective and relevant for these primary care physicians.[24]

The goal of our project was therefore to explore aspects of the consultation process/referral process between family physicians and geriatric psychiatrists. Specific objectives were to:

(1) Characterize the reasons seniors undergo psychogeriatric consultations.

(2) Describe expectations of the consultation visit for the patient/family, the referring family doctor, and the consultant.

(3) Assess patients' and family practitioners' level of satisfaction with the consultation process.

## Methods

St. Mary's Hospital Centre is a 300 bed, McGill University affiliated, Montreal community hospital well-suited to study seniors because it serves a catchment area 23% of whose population is 65 or older. Over an 18 month period all individuals 65 and older appearing at its Psychogeriatric Outpatient Clinic with family doctor-initiated consultations or referrals were approached by a research assistant to participate in this research ethics approved study.

Patients had to speak either English or French, or if they were unable, to be accompanied by a family member or informant who could. Verbal consent to screen for cognitive functioning was obtained since evidence of mental competency was required for written consent to participate in the study. On the SPMSQ scale [25] ranging from 0 (no impairment) to 10 (severe impairment) scores of 4 or less were satisfactory for informed consent for participation since they would be indicative, at most, of only mild cognitive impairment. Non-patient related exclusion criteria included referral from a specialist or a resident physician. The 3 members (with 3,12, and 25 years of practice respectively) of the Division of Psychogeriatrics of the Department of Psychiatry all participated.

Prior to the actual consultation the patient or family member/informant completed a pre-tested questionnaire on patient's age, gender, education, present or past occupation, marital status and place of residence. Immediately following the consultation the participants completed the Consultation Satisfaction Scale [26], an 18 item questionnaire that evaluates overall satisfaction with a medical visit, satisfaction with professional care, amount of time spent, and depth of the doctor-patient relationship.

Following each patient encounter the psychiatrist completed a short questionnaire on clinical diagnosis, recommendations made verbally to the patient, the written response to the referring family doctor, and perceptions on the reason for the consultation and expected process of care. These impressions were based on a composite of what was written on the consultation/ referral request and what was learned from the patient.

Following the consultation the research assistant notified the family doctor it had taken place and that the patient had given permission for the research team to contact him/her for additional information. To allow sufficient time for the referring physician to receive and evaluate the consultation report a subsequent 10 minute telephone interview with the family practitioner was scheduled to take place 4 weeks after the consultation.

The family practitioner was asked about the presence or absence of academic affiliation, type of practice (solo, group, hospital), frequency of referrals for psychogeriatric assessment; patient's diagnosis and treatment prior to the consultation; criteria for referral (e.g. diagnosis, treatment, investigation, reassurance, legal concern), and degree of pressure exerted by patient/family (on a 3 point scale) for the consultation [27]); whether the consult report had been received; patient's treatment at time of interview; whether the consult report met the expectations for which it had been made (ie was it for consultation, referral, or transfer of care); overall satisfaction with the consultation process; whether the physician would consult again; and suggestions for improvement in the overall consultation process. Additional physician demographic data was obtained from the directory of the College des Médecins du Québec.

After data collection from patients, psychiatrists, and family doctors was complete, the original consultation /referral notes from the latter were reviewed independently by a panel of one family physician (MJY) and two geriatric psychiatrists (FP,MC) to assess the reasons for the referrals. Since such notes tended to range from a few non-specific words to a detailed clinical history or specific queries (and sometimes legibility was an issue), the panel agreed in advance to categorize referrals according to clearly stated diagnoses (DSM IV) or constellations of symptoms.

## Results

### Patient participant characteristics

**Figure 1:**
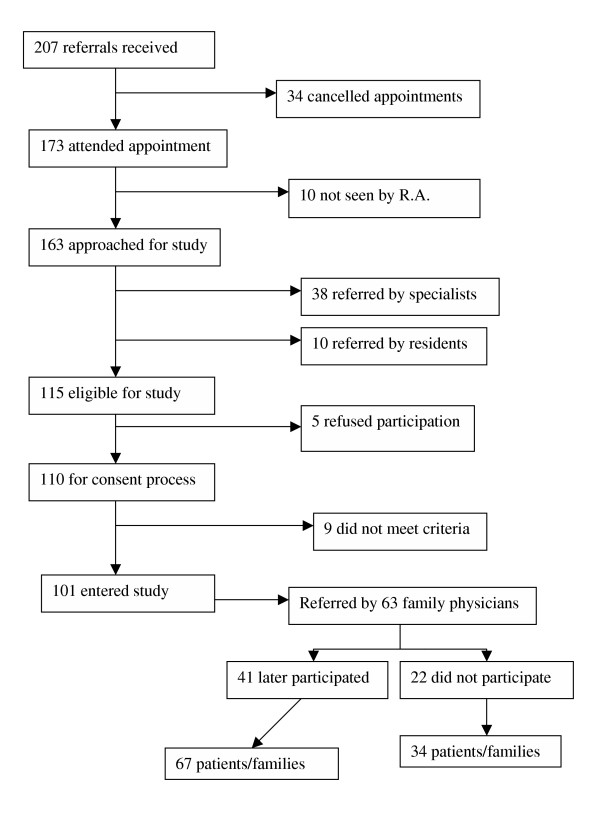
Identification of Patient/Family and Family Physician Samples

Over 18 months 207 people were referred by family practitioners for psychogeriatric consultation. As shown in Figure 1, 173 actually presented for the assessment and a research assistant was able to approach 163 of them about the study. With the application of exclusion and consent criteria and 5 eligible individuals refusing to participate, this number was reduced to 101. 82.2% (83/101) of them had SPMSQ scores suggestive of sufficient mental competency to complete the questionnaire while the remaining seniors required it be completed by another informant. The mean age of participants was 78.0, with 73.3% (74/101) female, just under half had a college or university education, and 79.3% having a past or current marriage. 71.3% of the sample were living in their own homes, 49.5% were living alone, 2/3 required assistance with housework and just under half needed help for food shopping or meal preparation. When patients were stratified according to the psychiatrist that they saw, no socio-demographic differences were found between the 3 groups. (data not shown) No demographic information was available for those patients who did not keep the consultation appointment.

### Reasons for consultation

The physician panel's blinded and independent review of reasons for the consultations showed full consensus for 76% (77/101) 44.6% (45/101) of the total were for depression, 31.7% (32 /101) for non-depressive problems such as anxiety or cognitive deficits, and in the remaining 24 % there was no consensus on 19 and insufficient information on which to judge 6. By comparison the frequency of psychiatrists' diagnoses were 47.5% (48/101) depression, 26.7% (27/101) dementia, anxiety 4% (4/101), bipolar disease 1% (1/101,)and 20.8 % (21/101) received any of 15 other diagnoses. For these 101 cases conditions the psychiatrists recommended medication in 85.1%, psychotherapy 19.8%, social work referral 10.9%, medical assessment/laboratory tests 14.9%, and other options in 9.9%.

### Participating family physician characteristics

The 101 consultation requests came from 63 family doctors, for whom follow-up was possible for 41 (65.1%) (Fig 1). The latter group was 55% male, had 18.3 mean years in practice, just over 40% were affiliated with our hospital, and were predominantly McGill University – trained. No statistically significant differences were found between these doctors and those who were not successfully contacted when compared for gender, years in practice, date and school of graduation, or hospital affiliation. As well, the patients coming from either group of physicians showed no clinically significant differences.

When contacted doctors were asked whether they believed that they had less, the same, or greater interest in psychiatry as compared to the average family doctor they were equally divided between the latter two options. To support this perception 60% had attended a CME event on a psychiatry topic in the 3 years preceding the study. In the 12 months preceding the study the frequency these doctors initiated consultations with psychiatrists was 65.0% (26/41) for 1–5 patients, 27.5% (11/41) for 6 – 10, and 7.5% (3/41) for more than 10. These doctors indicated however that prior to initiating their referrals they attempted in 75.8% (50/67) of cases to diagnose and manage the patients on their own and in 46% this took place for greater than 6 months.

### Consultation for management strategy

For the sample of 67 patients (Fig 1) Table 1 suggests that the primary motivations behind the consultations for both referring and consulting doctors were diagnosis and management concerns. At face value the differences in the proportions for the latter appear to be wide between the two groups of doctors (62.7% vs. 48.5% respectively). However, grouping potential options with possible overlapping meanings – e.g. management strategy, failed treatment, dealing with treatment side effects, and reassurance – psychiatrists and family doctors actually had similar perceptions on management concerns (65.7% vs. 60.6% respectively).

**Table 1:** Perceived reason for consultation

	(N = 67)			
	By Psychiatrist		By Family Physician	
	N	%	N	%
Diagnosis	15	22.4	20	30.3
Management strategy	42	62.7	32	48.5
Patient/family request	2	3	3	4.6
Lack of skill/facilities to treat	2	3	2	3
Failed treatment	2	3	5	7.6
Reassurance	0	0	2	3
Medico-legal	1	1.5	0	0
Treatment side-effect	0	0	1	1.5
Follow-up	2	3	0	0
Other	0	0	1	1.5
Don't know	1	1.5	0	0
				

Total	67	100	66*	100

*excludes missing values

### Perceptions about process of care

For the sample of 67 patients (Fig 1) the family practitioners reported that for 72.3% of referrals they indicated to the patient or family member what to expect as a result of the consultation visit. Nonetheless, there was wide variation between the family doctors, psychiatrists, and patients as to perception of the process of assessment and follow-up (Table 2). Psychiatrists saw the referral as a request for only an assessment for 80.6% of the patients, while family doctors expected short to long-term care by the psychiatrists in 55.4% of cases. Patients/families showed a large range of expectation, and about half actually had no expectation or opinion. When such analyses were done for the pairing of 101 patients/families with psychiatrists comparable findings were found. Finally, looking at manpower utilization appropriate to implement consultation recommendations, little difference between the 2 doctor groups was found, and use of nurses or community resources seemed to not be considered often. (Table 3)

**Table 2:** Expectations of Process of Care

	(N = 67)					
	Psychiatrist		FP*		Patient/Informant	
						
	N	%	N	%	N	%
Assess and return patient to FP	54	80.6	28	43.1	16	24.2
Assess, treat short term, and return to FP	7	10.4	34	52.3	4	6.1
Assess, transfer care to psychiatrist	3	4.5	2	3.1	12	18.2
Other	1	1.5	1	1.5	2	3
No expectation	0	0	0	0	21	31.8
Don't know	2	3	0	0	11	16.7
						

Total	67	100	65**	100	66**	100

*FP = Family physician

**excludes missing values

**Table 3:** Expectations of who would implement recommendations

	(N = 67)			
	Psychiatrist		Family Physician	
				
	N	%	N	%
Referring physician	35	53.9	32	54.2
Consulting psychiatrist	24	36.9	18	30.5
Both	3	4.6	1	1.7
Geriatric psychiatry nurse	1	1.5	0	0
Community Resource	1	1.5	0	0
Unclear	1	1.5	8	13.6

Total	65*	100	59*	100

*Excludes missing values

Table 4 examines concordance between pairings of patients, family doctors and psychiatrists for 3 aspects of care. On "expectations of process of care" family doctors and psychiatrists agreed as often as they disagreed; patients and psychiatrists disagreed 3/4 of the time, and patients and family doctors disagreed almost 90% of the time. On "reason for the consultation" family doctors and psychiatrists once again agreed as often as they disagreed, and only for "identification of the professional responsible for follow-up" was the tendency for agreement found to be strongest. Interestingly, despite such differences Table 5 suggests that patients/ informants were nonetheless generally satisfied with the care, depth, and length of the consultation. Patients' / informants' satisfaction was found not to be associated with whether they and their family doctors were concordant on the process of care. Similarly, family physicians' satisfaction was not associated with whether they and the psychiatrists had concordance on the process of care, reason for the consultation, or responsibility for follow-up. (Table 6)

**Table 4:** Concordance: Family physician, psychiatrist, patient

	Total cases = 67			
	Agree		Not agree	
	N	%	N	%
Reason for consultation				
Family physician and psychiatrist	33	49.3	34	50.7
				
				
Expectations of process of care				
Patient and Family Physician	9	13.4	58	86.6
Patient and Psychiatrist	15	22.4	52	77.6
Family physician and psychiatrist	32	47.8	35	52.2
				
				
Professional responsible for implementing recommendations				
				
Family physician and psychiatrist	41	61.2	26	38.8

**Table 5:** Patient/Informant Satisfaction* with Consultation

	N = 101**	
	Mean	(std dev)
General Satisfaction*	4.11	(0.77)
Professional Care	4.06	(0.53)
Depth of relationship	3.64	(0.69)
Length of Consultation	3.67	(0.97)

* Satisfaction ranged from 1 (very dissatisfied) to 5 (very satisfied)

** 7 missing

**Table 6:** Impact of Expectations and Concordance on Satisfaction*

Patients'/Informants' Expectations					
	FP/Patient Concordance		FP/Patient Non-concordance		
					
	Mean	(std dev)	Mean	(std dev)	t-test(p-value)
	
On Process of care	4	(0.5)	3.8	(0.6)	0.300
					
Physicians' Expectations					
	FP/Patient Concordance		FP/Patient Non-concordance		
	Mean	(std dev)	Mean	(std dev)	t-test(p-value)
	
On Process of care	4.1	(1.0)	4.2	(0.8)	0.616
On reason for consultation	4.1	(1.0)	4.2	(0.8)	0.787
On follow-up after consultation	4.3	(0.8)	4.0	(1.1)	0.178

*Satisfaction ranged from 1 (very unsatisfied) to 5 (very satisfied)

** FP = family physiciana

### Family doctors' assessment of consultation

The family doctors acknowledged receiving psychiatrists' consultation reports from the psychiatrists in 91 % (61/67) of cases. They rated the overall consultation process as satisfactory to very satisfactory 83.3% of the time. 67.2% (41/61) of consult reports were felt to be very useful, compared with 27.9% (17/61) perceived as somewhat useful. The reports' information was rated good to very good in 74.3% of cases. Consultants' recommendations were felt to be clear in just over 83%. Two thirds of FPs had no comments or recommendations on how to improve the consultation/referral process. Among those making suggestions the most frequent was a request for typed consultation reports.

## Discussion

### Depression as a common problem

This study examined aspects of the consultation/referral process for seniors from family practitioners to geriatric psychiatrists. The predominant diagnosis for which help was sought was depression, a diagnosis not unexpected given the relatively high prevalence and multi-faceted etiology of depression in the elderly (medication, co-morbidity, loss/grieving, disability).

### Uncertainty about management

When one looks at referrals to sub-specialized mental health services (e.g. a cultural consultation service), the vast majority are reported to be made for diagnostic purposes.[28] Pressure from patients and/or families has been hypothesized as a cause for initiating consultation in internal medicine care.[29] Our data, however, would suggest that within the specific sample studied this does not seem to be an important factor. A recent study looking at referral patterns from generalists to specialists (that did not include psychiatrists) found that therapy management was the most common reason for referral.[29] In our study, from the perspectives of both family doctors and psychiatrists the predominant reason for referral was for assistance with management strategies. Since 3/4 of the cases had management attempts by the family practitioner prior to referral (almost half for greater than 6 months) one wonders if this reflects specific challenges in the care of mental health problems in seniors. Alternatively, since the consulting psychiatrists felt 85.1% of referrals needed pharmacotherapy, family physicians may be having trouble in this domain – either in knowledge base, in how to choose the right drug and evaluate its efficacy, or in how to changes drugs.

### Post-consultation care

About 1/2 as many patients as family doctors, and similarly 1/2 as many of family doctors as psychiatrists had an expectation for the consultant to assess and return the patient to the referring physician for care. The psychiatrists' viewpoint may derive from a desire to limit their roles specifically to that of consultants (not to primary therapists), consistent with evolving consensus on roles for psychiatrists [9,10]. Alternatively, practicing in a catchment area with a high proportion of seniors, they may have felt a practical necessity to rapidly return patients to the family physicians. On the other hand, since 75.8 % of family physicians indicated that they had tried diagnosis and management prior to referral, they may be indicating insufficient comfort or experience with their roles as the major care providers for seniors with mental health problems. This may generate a preference for the security of having the psychiatrists initiate treatment and follow for at least a short time. This observation may be important since, at least on the basis of years in practice, the responding cohort of physicians was an experienced one. Nonetheless, the proportionately equal agreement between the two professions as to who should implement recommendations from consultations suggests a willingness by family doctors to assume subsequent care of agreed upon patients.

### Communication problems

The study does suggest that family doctors likely have to do a better job of explaining their consultation expectations to both patients/families and consultants, and that psychiatrists likely need to examine their communication on this, as well. Since the majority of referring doctors had sent fewer than 5 patients in the preceding 12 months for psychiatric consultation one wonders if the low concordance between the psychiatrists and the family doctors reflects the need for a threshold number of consultations to promote different or more specific communication between referring physician and consultant.

In just under 3/4 of the referrals the family doctors indicated that they told their patients what the process of care likely would be. Nonetheless 48.5% of patients/families indicated they had no expectation or did not know what to expect from the consultation visit. Interestingly, a larger proportion of patients, when compared to either group of doctors, expected transfer of care to the psychiatrists. Again this raises questions of how well the referring physician prepared the patient for the consultation, or were some patients so distressed by symptoms or the idea of having to see a psychiatrist that they did not hear or retain what they were told? Alternatively, is there something in the community mindset of mental health care that suggests seeing a psychiatrist naturally implies engaging in some form of on-going therapy?

### Study limitations

Conclusions are restricted to those seniors with mental health problems who kept their appointments for a psychogeriatric consultation. The study may also be limited by its predominantly descriptive nature and by the relatively low family doctor participation rate in the follow-up survey. Although general characteristics of doctors were similar, it is possible that those who were less satisfied and perhaps less concordant, did not participate. Further, since the interview with family practitioners was retrospective, the information on, for example, motive for consulting, might have been distorted by recall or wishful thinking.

## Conclusion

This study appears to have identified some concerns about the process and possible outcome of consultation/referral for mental health care for seniors. Our findings support Canadian recommendations towards the development of new models of shared care for mental health [9,10] and parallel observations made in internal medicine about the need for different communication patterns for consultations.[29]

One solution may lie in a pre-consultation orientation to patient /family (in verbal, written, or videotape form) about what the visit may entail and aim to achieve. Another sees an expanded role for nurses or other appropriately trained individuals to function as comprehensive case managers. [15-20] Further, the consultation and referral process might be improved through the use of consultation request and response forms that specifically structure written content according to mutually pre-determined headings.

## Competing interests

The author(s) declare that they have no competing interests.

## Authors' contributions

FP, JM, MJY, MC, and JL made contributions to the study conception and design; MJY, FP, MC, ME, and JL were involved in acquisition of data; ND, EB, MJY, JM and FP were involved in data analysis; MJY, FP, JM, and MC were involved in drafting the article; and all authors have had critical input and have read and approved the article.

## Pre-publication history

The pre-publication history for this paper can be accessed here:


